# Using Human ‘Personalized’ Cybrids to Identify Drugs/Agents That Can Regulate Chronic Lymphoblastic Leukemia Mitochondrial Dysfunction

**DOI:** 10.3390/ijms241311025

**Published:** 2023-07-03

**Authors:** Lata Singh, Shari Atilano, Marilyn Chwa, Mithalesh K. Singh, Mustafa Ozgul, Anthony Nesburn, M. Cristina Kenney

**Affiliations:** 1 Department of Ophthalmology, Gavin Herbert Eye Institute, University of California Irvine, Irvine, CA 92697, USA; drlata.singh@aiims.edu (L.S.); satilano@hs.uci.edu (S.A.); mchwa@hs.uci.edu (M.C.); mithales@hs.uci.edu (M.K.S.); ozgulm@hs.uci.edu (M.O.); anesburn@hs.uci.edu (A.N.); 2 Department of Pediatrics, All India Institute of Medical Institute, New Delhi 110029, India; 3 Department of Pathology and Laboratory Medicine, University of California Irvine, Irvine, CA 92697, USA

**Keywords:** chronic lymphoblastic leukemia, cybrid, mitochondria, nutraceutical, ibrutinib

## Abstract

This study uses personalized chronic lymphoblastic leukemia (CLL) cybrid cells to test various drugs/agents designed to improve mitochondrial function and cell longevity. Age-matched control (NL) and CLL cybrids were created. The NL and CLL cybrids were treated with ibrutinib (Ibr-10 μM), mitochondrial-targeted nutraceuticals such as alpha lipoic acid (ALA-1 mM), amla (Aml-300 μg), melatonin (Mel-1 mM), resveratrol (Res-100 μM) alone, or a combination of ibrutinib with nutraceuticals (Ibr + ALA, Ibr + Aml, Ibr + Mel, or Ibr + Res) for 48 h. MTT (3-(4,5-dimethylthiazolyl-2)-2,5-diphenyltetrazoliumbromide), H2DCFDA(2′,7′ Dichlorodihydrofluorescein diacetate), and JC1 assays were used to measure the cellular metabolism, intracellular ROS levels, and mitochondrial membrane potential (∆ψm), respectively. The expression levels of genes associated with antioxidant enzymes (*SOD2*, *GPX3*, and *NOX4*), apoptosis (*BAX* and *CASP3*), and inflammation (*IL6*, *IL-1β*, *TNFα*, and *TGFβ*) were measured using quantitative real-time PCR (qRT-PCR). CLL cybrids treated with Ibr + ALA, Ibr + Aml, Ibr + Mel, and Ibr + Res had (a) reduced cell survivability, (b) increased ROS production, (c) increased ∆ψm levels, (d) decreased antioxidant gene expression levels, and (e) increased apoptotic and inflammatory genes in CLL cybrids when compared with ibrutinib-alone-treated CLL cybrids. Our findings show that the addition of nutraceuticals makes the CLL cybrids more pro-apoptotic with decreased cell survival compared with CLL cybrids exposed to ibrutinib alone.

## 1. Introduction

Chronic lymphocytic leukemia (CLL) is the most common adult leukemia in the western world, accounting for approximately 25% of adult leukemia. The incidence of CLL in the US is 4.5 cases per 100,000 people, and the median age at the diagnosis is 71 years. The incidence of CLL is higher in individuals with family history, of Caucasian ethnicity, and with Agent Orange exposure [1,2]. First-line therapy typically consists of nucleoside analogues (fludarabine) and alkylating agents (cyclophosphamide, chlorambucil, and bendamustine) in combination with monoclonal antibodies directed against CD20 (rituximab) [3]. Targeted agents, including tyrosine kinase inhibitors, such as ibrutinib (BTK-Bruton’s tyrosine kinase inhibitor) and idelalisib (PI3Kδ-phosphoinositide-3-kinase inhibitor), along with small molecules such as venetoclax (B-cell lymphoma 2 gene inhibitor), are currently available in the relapse setting [4]. 

Cancer is characterized by altered cellular metabolism, which is increasingly being targeted for therapeutic intervention [4]. Cell death, cell differentiation, innate immunity, hypoxia sensing, calcium metabolism, amino acid and iron sulfur cluster metabolism, and heme biosynthesis all rely on mitochondria [5]. CLL cells have extremely active mitochondria that consume high levels of oxygen leading to elevated electron transport chain activities and ATP production through the oxidative phosphorylation (OXPHOS) pathway rather than aerobic glycolysis [6,7]. The CLL mitochondria have increased mass, membrane potential, and DNA copy numbers indicating higher numbers of mitochondria per cell than normal [8].

Respiratory insufficiency is the origin of cancer according to Warburg’s theory. All other phenotypes of the disease, including the somatic mutations, arise either directly or indirectly from insufficient respiration [9,10,11]. The theories of C.D. Darlington and others, which demonstrate that cancer is primarily a mitochondrial disease of the cytoplasm, were consistent with Warburg’s metabolic theory [12,13,14]. 

Excessive fatigue and other common side effects of chemotherapy treatments can result from mitochondrial dysfunction, the key organelle responsible for cellular energy production. As a result, the efficiency of OXPHOS is reduced, as is the production of adenosine-5’-triphosphate (ATP). Several components of OXPHOS require routine replacement, which can be aided by mitochondria-targeted nutraceuticals [15]. These include vitamins, minerals, antioxidants, metabolites, enzyme inhibitors and cofactors, mitochondrial transporters, herbs, membrane phospholipids (i.e., alpha lipoic acid (ALA)), amla, melatonin, and resveratrol [15,16]. 

Transmitochondrial cybrids (cell lines with identical nuclei but mitochondria from different individuals) are one approach to elucidating the functional consequences of possessing mtDNA from healthy subjects versus subjects with diseases [17,18]. Furthermore, the cybrid model is excellent to characterize the molecular and biological responses of an individual’s mitochondria to specific exogenous treatment regimens [14].

In this study, we used CLL patient-derived cybrids to determine whether mitochondrial dysfunction contributed to ibrutinib resistance and whether ibrutinib combined with a mitochondria-targeting nutraceutical could mitigate this chemoresistance.

## 2. Results

### 2.1. Sequencing of mtDNA from Patient-Derived Cybrid from Chronic Lymphoblastic Leukemia (CLL)

The entire mtDNA from the CLL cybrid was sequenced using Sanger sequencing. The CLL cybrid was sequenced to identify the SNPs that defined the individual’s mtDNA haplogroup. The CLL cybrid demonstrated homoplasmic SNPs that represented the N1b1b1 haplogroup (Supplementary Table S1). In this haplogroup profile, there were nine non-synonymous SNPs, which led to the following amino acid changes: m.4735C>A, Thr:Asn89; m.4917A>G, Asn:Asp150; m.4960C>T, Ala:Val164; m.8472C>T, Pro:Leu36; m.8836A>G, Met:Val104; m.11928A>G, Asn:Ser390; m.12705C>A, Ile:Met123; m.14766C>T, Thr:Ala194; and m.15326A>G, Thr:Ala194.

The unique SNPs were not listed in www.hmtvar.uniba.it (accessed on 12 October 2022) or other platforms. Table S1 shows the SNPs in the CLL cybrid. There were two private SNPS (those that do not define the haplogroup) in the mtDNA regions of the CLL cybrid: m.2866A>T (no rs#, MT-RNR2) and m.13635T>C (no rs#, MT-ND5). The Sanger sequencing methodology allowed the identification of heteroplasmic SNPs in the CLL cybrid. The mtDNA in CLL cybrid contained six heteroplasmy SNPs, which are listed as follows: m.1598G>A (rs3135027, MT-RNR1); m.1703C>T (rs28527344, MT-RNR2); m.1719G>A (rs3928305, MT-RNR2); m8836A>G (no rs#, MT-ATP6); m.11928A>G (no rs#, MT-ND4); and m.16390G>A (rs4137895, MT-DLOOP).

Association with Pathogenesis: The m.73A>G, m.152T>C, m.16223C>T, and m.16390G>A SNPs located in the MT-DLOOP region and the m.14766 C>T and m.15326 A>G in MT-CYB region have been reported in other cancer types, mitochondrial disease, and normal tension glaucoma (NTG) [19,20,21,22,23].

### 2.2. CLL Patient-Derived Cybrid with Dysfunctional Mitochondria Exhibits Resistance to Ibrutinib Treatment

**Figure 1:**
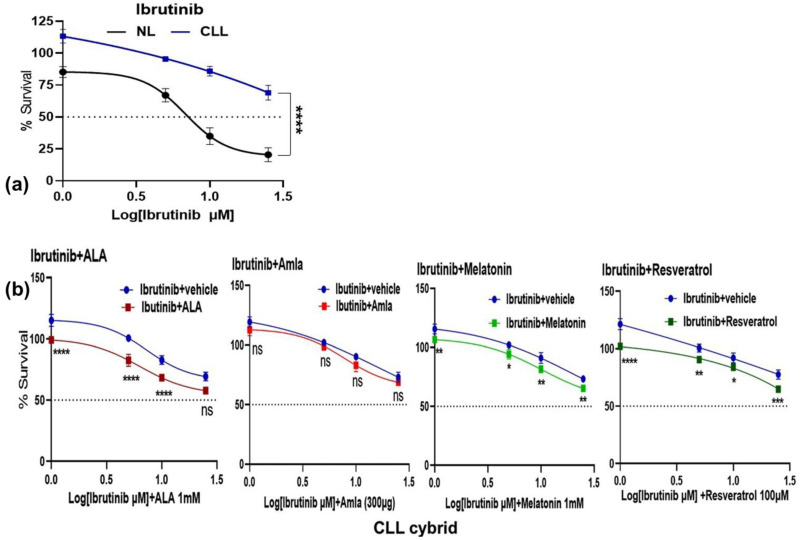
Effect of individual ibrutinib (Ibr) and (Ibr) in-combination treatment with mitochondria-targeted nutraceuticals, i.e., alpha lipoic acid (ALA), amla (Aml), melatonin (Mel), and resveratrol (Res) {Ibr + ALA; Ibr + Aml; Ibr + Mel; Ibr + Res} on the survival of the cybrid derived from age-matched control (NL) and chronic lymphoblastic leukemia (CLL) patient. (**a**) The effect of ibrutinib treatment on the survival of NL and CLL cybrids using the MTT assay. (**b**) MTT assay of the effects of Ibr + ALA, Ibr + Aml, Ibr + Mel, and Ibr + Res treatments on the survival of CLL cybrids. One-way ANOVA was used to analyze the data shown as mean SD. ns denotes non-significant, * denotes *p*-value 0.05; ** 0.01; *** 0.001; **** 0.0001.

The mitochondria, an essential intracellular signaling core, are emerging as key determinants of a wide range of features central to cancer’s initiation, development, and progression, including metabolic reprogramming, metastasis, and chemotherapeutic resistance [24]. First, we assessed if mitochondrial dysfunction was linked to the development of resistance to ibrutinib treatment in this CLL patient. To test this hypothesis, we created patient-derived cybrid cell lines using platelets collected from an age-matched control (NL) individual and a CLL patient who was not responding to ibrutinib treatment. As shown in Figure 1a, treatment with ibrutinib decreased the survival of the NL cybrid in a dose-dependent manner, as expected. While the CLL cybrid cells were significantly resistant to ibrutinib treatment compared with the NL cybrid at the 1.5 µM concentration. This finding indicated that mitochondrial dysfunction diminished the response to the ibrutinib treatment by increasing resistance in the CLL patient.

Next we asked whether individual treatment with mitochondria-targeting nutraceuticals such as alpha lipoic acid (ALA) [25], amla [26,27], melatonin [28], and resveratrol [29] or treatment in combination with ibrutinib could overcome the resistance in the CLL patient-derived cybrid. In the absence of ibrutinib, we determined the optimal concentratTion of each nutraceutical in NL and CLL patient-derived cybrids required to promote cell viability, and the concentrations of ALA (1 mM), amla (300 μg), melatonin (1 mM), and resveratrol (100 μM) were chosen (Supplementary Figure S1). Then, in the CLL cybrid, we tested the combination treatment of ibrutinib with the optimal concentration of mitochondrial-targeting nutraceuticals. As shown in Figure 1b, when compared with ibrutinib treatment alone, ibrutinib combination treatment with ALA, amla, melatonin, and resveratrol reduced the mean survival rate in the CLL cybrid by 15% (*p* < 0.0001), 5% (*p* = 0.095), 8% (*p* = 0.008), and 12% (*p* < 0.0001), respectively. The addition of ALA and resveratrol enhanced the response to ibrutinib in CLL cybrids (lower percent survival) compared with the response found in co-cultures of melatonin plus ibrutinib. Amla had no significant effect on the ibrutinib response in the CLL cybrid. Our findings suggest that these mitochondria-targeted nutraceuticals lowered the survivability of the CLL cybrids exposed to ibrutinib. We then tested the hypothesis that combining the ibrutinib with the nutraceuticals may promote the levels of ROS production and apoptosis.

### 2.3. Mitochondria-Targeted Nutraceutical and Ibrutinib Treatment Together Increases Total ROS Production in a CLL Patient-Derived Cybrid

**Figure 2:**
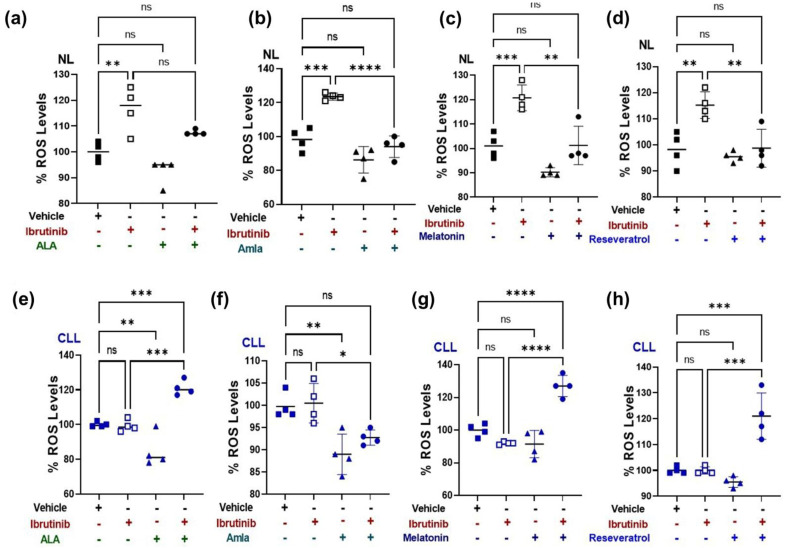
Effect of ibrutinib (Ibr), alpha lipoic acid (ALA), amla (Aml), melatonin (Mel), resveratrol (Res), and in-combination treatment with mitochondrial-targeted nutraceuticals, i.e., {Ibr + ALA; Ibr + Aml; Ibr + Mel; Ibr + Res}, on the reactive oxygen species (ROS) levels produced by age-matched control (NL) and chronic lymphoblastic leukemia (CLL) cybrids. Using H_2_DCFDA (2,7-dichlorodihydrofluorescin diacetate) assay: (**a**) ROS measured on the treatment with ibrutinib Ibr alone, ALA alone, and Ibr + ALA on the NL cybrid. (**b**) ROS measured on the treatment with Ibr alone, Aml alone, and Ibr + Aml on the NL cybrid. Data are shown as percent ROS of vehicle control. (**c**) ROS measured on the treatment with Ibr alone, Mel alone, and Ibr + Mel on the NL cybrid. (**d**) ROS measured on the treatment with Ibr alone, Res alone, and Ibr + Res on the NL cybrid. Data are shown as percent ROS of vehicle control. (**e**) ROS measured on the treatment with Ibr alone, ALA alone, and Ibr + ALA on the CLL cybrid. (**f**) ROS measured on the treatment with ibrutinib (Ibr), amla (Aml), and Ibr + Aml on the CLL cybrid. Data are shown as percent ROS of vehicle control. (**g**) ROS measured on the treatment with Ibr alone, Mel alone, and Ibr + Mel on the CLL cybrid. Data are shown as percent ROS of vehicle control. (**h**) ROS measured on the treatment with Ibr alone, Res alone, and Ibr + Res on the CLL cybrid. Data are shown as percent ROS of vehicle control. Data shown as mean ± SD were analyzed by one-way ANOVA test. ns represents non-significant * indicates *p*-value ≤ 0.05; ** *<* 0.01; *** *<* 0.001; **** *<* 0.0001.

ROS production has been linked to chemotherapy responses via its effects on cell survival or death signaling cascades [30,31,32]. This has led to speculation that ROS modulators could be used to prevent cancer or improve therapeutic responses [33]. Next, we wanted to see if combining a mitochondria-targeting nutraceutical (ALA, amla, melatonin, and resveratrol) in combination with ibrutinib treatment raised the level of ROS production more than ibrutinib treatment alone. As shown in Figure 2a, treatment with ibrutinib (10 μM) for 48 h increased the mean difference levels of ROS production in the NL cybrid by 16% (*p* = 0.003) but had no effect on ROS levels in the CLL cybrid (Figure 2e). In the NL cybrid cells, treatment with ALA, amla, melatonin, and resveratrol alone did not affect the ROS levels (Figure 2a–d). In the NL cybrids, when the ibrutinib treatment was combined with ALA (1 mM), amla (300 μg), melatonin (1 mM), and resveratrol (100 μM), there was no change in the mean difference of ROS levels in Ibr + ALA (+7.5%, *p* = 0.256; Figure 2a), Ibr + amla (−4%, *p* = 0.817; Figure 2b), Ibt + Mel (+0.25%, *p* = 0.999; Figure 2c), and Ibr + Res (+0.5%, *p* = 0.999; Figure 2d), respectively, compared with the vehicle control-treated cells. However, in the NL cybrid compared with ibrutinib alone, there was a significant decrease in ROS levels when ibrutinib was combined with amla (Ibr + Amla, −29%, *p* < 0.0001), melatonin (Ibr + Mel, −19%, *p =* 0.001), and resveratrol (Ibr + Resv, −16%, *p* = 0.005) (Figure 2b–d).

In the CLL cybrids, there were increased levels of ROS by 21% (Ibr + ALA, *p* = 0.0007; Figure 2e), 7% (Ibr + Amla, *p* = 0.067; Figure 2f), 27% (Ibr + Mel, *p* < 0.0001; Figure 2g), and 21% (Ibr + Resv, *p* = 0.0002; Figure 2h) compared with the vehicle control. In the CLL cybrids, when ibrutinib was combined with ALA (+22%, *p* = 0.0005; Figure 2e), melatonin (+35%, *p* < 0.0001; Figure 2g), or resveratrol (+21%, *p* = 0.0002; Figure 2h), there were significantly higher levels of ROS compared with ibrutinib-alone-treated CLL cybrids. 

These findings support that in CLL cybrids, the combined treatments of ibrutinib plus ALA, melatonin, or resveratrol have increased ROS production, which may sensitize the CLL cybrids to lower cell survival compared with ibrutinib treatment alone. 

### 2.4. Mitochondrial Membrane Potential (ΔΨm) Increases with Mitochondria Targeted Nutraceutical and Ibrutinib Combination Treatment in a CLL Patient-Derived Cybrid

**Figure 3:**
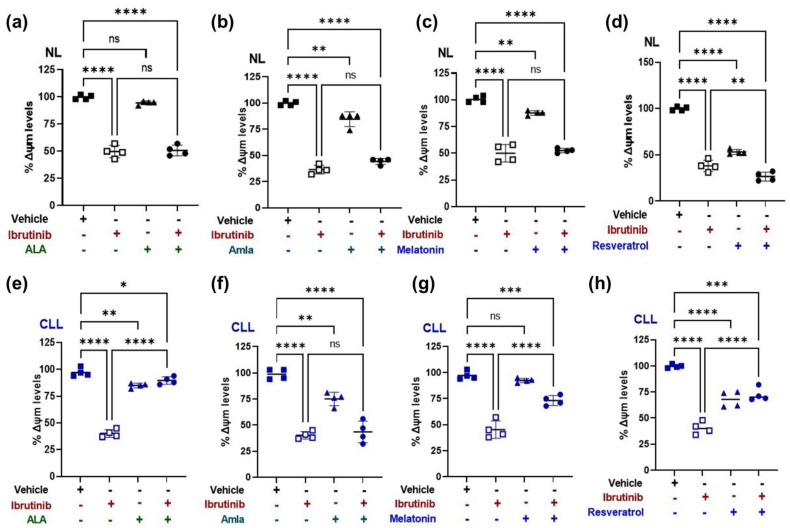
Effect of ibrutinib (Ibr), alpha lipoic acid (ALA), amla (Aml), melatonin (Mel), resveratrol (Res), and in-combination treatment with mitochondria-targeted nutraceuticals, i.e., {Ibr + ALA; Ibr + Aml; Ibr + Mel; Ibr + Res}, on the mitochondrial membrane potential (ΔΨm) cybrid derived from age-matched control (NL) and chronic lymphoblastic leukemia (CLL) cybrids. Using JC1 assay: (**a**) ΔΨm measured on the treatment with ibrutinib (Ibr), alpha lipoic acid (ALA), and Ibr + ALA on the NL cybrid. Data are shown as percent ΔΨm of vehicle control. (**b**) ΔΨm measured on the treatment with Ibr alone), Aml alone, and Ibr + Aml on the NL cybrid. Data are shown as percent ΔΨm of vehicle control. (**c**) ΔΨm measured on the treatment with Ibr alone, Mel alone, and Ibr + Mel on the NL cybrid. Data are shown as percent ΔΨm of vehicle control. (**d**) ΔΨm measured on the treatment with Ibr alone, Res alone, and Ibr + Res on the NL cybrid. Data are shown as percent ΔΨm of vehicle control. (**e**) ΔΨm measured on the treatment with Ibr alone, ALA alone, and Ibr + ALA on the CLL cybrid. Data are shown as percent ΔΨm of vehicle control. (**f**) ΔΨm measured on the treatment with Ibr alone, Aml alone, and Ibr + Aml on the CLL cybrid. Data are shown as percent ΔΨm of vehicle control. (**g**) ΔΨm measured on the treatment with Ibr alone, Mel alone, and Ibr + Mel on the CLL cybrid. Data are shown as percent ΔΨm of vehicle control. (**h**) ΔΨm measured on the treatment with Ibr alone, Res alone, and Ibr + Res on the CLL cybrid. Data are shown as percent ΔΨm of vehicle control. Data shown as mean ± SD were analyzed by one-way ANOVA test. ns represents non-significant * indicates *p*-value *≤* 0.05; ** *<* 0.01; *** *<* 0.001; **** *<* 0.0001.

Next, we wanted to evaluate whether combining mitochondria-targeting nutraceuticals (ALA, amla, melatonin, and resveratrol) with ibrutinib raises or lowered the levels of mitochondrial membrane potential (ΔΨm) more than ibrutinib alone. Ibrutinib (10 μM) treatment for 48 h reduced the mean difference of mitochondrial membrane potential (ΔΨm) levels in both the NL cybrid (50%, *p* < 0.0001; Figure 3a–d) and the CLL cybrid (57%, *p* < 0.0001; Figure 3e–h) compared with the vehicle control. Compared with the vehicle-alone treatment, the ibrutinib treatment in combination with ALA, amla, melatonin, and resveratrol in the NL cybrid reduced the mean difference of ΔΨm levels by 49% (Ibr + ALA, *p* < 0.0001), 55% (Ibr + Amla, *p* < 0.0001), 48% (Ibr + Mel, *p* < 0.0001), and 73% (Irb + Resv, *p* < 0.0001) (Figure 3a–d). 

In the NL cybrids, when compared with the ibrutinib-alone treatment, the ibrutinib treatment plus supplement did not change the mean difference of the ΔΨm levels in ALA (Ibr + ALA, *p* = 0.994; Figure 3a), amla (Ibr + Aml, 7.7%, *p* = 0.112; Figure 3b), or melatonin (Ibr + Mel, 2.5%, *p* = 0.912; Figure 3c). However, when combined with resveratrol (Ibr + Resv), the ΔΨm level was reduced by 32% (*p* < 0.0001; Figure 3d) compared with the ibrutinib-alone treatment. 

In CLL cybrids the ibrutinib treatment alone decreased ΔΨm levels by 57% (*p* < 0.0001; Figure 3e–h) compared with the vehicle-treated CLL cybrids. In the CLL cybrids treated with ibrutinib plus ALA, amla, melatonin, and resveratrol, there were reduced mean difference levels of ΔΨm compared with the vehicle control by 7% (Ibr + ALA, *p* = 0.034), 55% (Ibr + Amla, *p* < 0.0001), 24% (Ibr + Mel, *p* = 0.0001), and 27% (Irb + Resv, *p* = 0.0001), respectively. Interestingly, when ibrutinib was combined with ALA (+49%, *p* < 0.0001; Figure 3e), melatonin (+28%, *p* < 0.0001; Figure 3g), or resveratrol (+32%, *p* < 0.0001: Figure 3h), there were significantly higher levels of ΔΨm in CLL cybrids compared with ibrutinib-alone-treated CLL cybrids. These findings implied that ibrutinib alone reduced ΔΨm levels in CLL cybrids more than the in-combination treatment.

### 2.5. Effect of Ibrutinib on the Expression of Antioxidant, Apoptotic, and Inflammatory Genes in the Patient-Derived CLL and NL Cybrids

**Figure 4:**
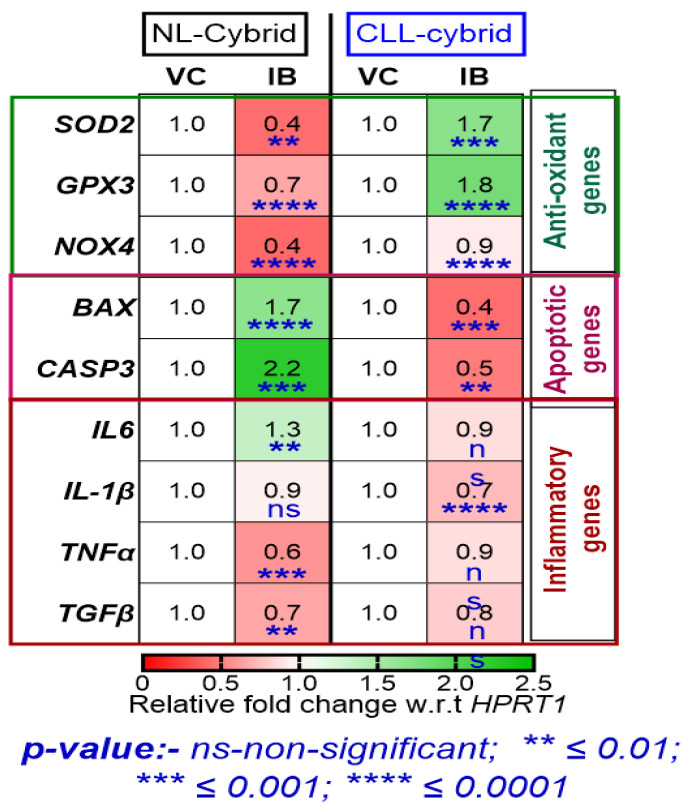
Heat map representation of treatment with ibrutinib (Ibr) on the mRNA expression of genes related to antioxidant, apoptotic, and inflammation using quantitative real-time polymerase chain reaction (qPCR) in the cybrid derived from age-matched control (NL) and chronic lymphoblastic leukemia (CLL) cybrids. Data are shown in the form of relative fold change with respect to housekeeping gene HPRT1 with respect to vehicle control and were analyzed by one-way ANOVA test. ns represents non-significant, *p*-value ** *<* 0.01; *** *<* 0.001; **** *<* 0.0001.

Antioxidant gene expression: As shown in Figure 4, when cybrids were treated with ibrutinib (10 μM), there was a significant decrease in the relative fold change of *SOD2* (0.4-fold change, *p* < 0.01), *GPX3* (0.7-fold change, *p* < 0.0001), and *NOX4* (0.4-fold change, *p* < 0.0001) in the NL cybrid. In contrast, in the CLL cybrids, treatment with ibrutinib alone caused a significant increase in the relative fold change of *SOD2* (1.7-fold change, *p* < 0.01), *GPX3* (1.8-fold change, *p* < 0.0001), and *NOX4* (0.9-fold change, *p* < 0.0001) expression. These results suggest that the resistance phenomena that developed against ibrutinib treatment in the CLL cybrid may also have been due to the increased expression of antioxidant, which might have diminished the production of ROS as shown in Figure 2e.

Apoptotic gene expression: Next, we wanted to investigate the effect of ibrutinib on the expression of genes related to apoptosis in the CLL and NL cybrids. Treatment with ibrutinib (10 μM) resulted in a significant increase in the relative fold change of *BAX* (fold change = 1.7, *p* < 0.0001) and *CASP3* (fold change = 2.2, *p* < 0.001) in the NL cybrid (Figure 4). In contrast, ibrutinib treatment of the CLL cybrid caused a significant decrease in the relative fold change of *BAX* (fold change = −0.4, *p* < 0.0001) and *CASP3* (fold change = −0.5, *p* < 0.01) expression (Figure 4).

Inflammatory gene expression: Treatment of NL cybrids with ibrutinib resulted in a significant increase in the mean fold change of *IL-6* (1.3-fold change, *p* < 0.01) but no change in the expression of *IL1β*, while there was a significant decrease in the mean fold change of *TNFα* (0.6-fold change, *p* < 0.001) and *TGFβ* (0.7-fold change, *p* < 0.01). In contrast, ibrutinib treatment of the CLL cybrid caused a significant decrease in the mean-difference fold change of *IL1β* (*p* < 0.0001), whereas it had no significant effect on the expression of other inflammatory genes (Figure 4).

### 2.6. Effect of Mitochondrial Targeted Nutraceuticals on the Expression of Antioxidant, Apoptotic, and Inflammatory Genes in the Patient-Derived CLL and NL Cybrids

**Figure 5:**
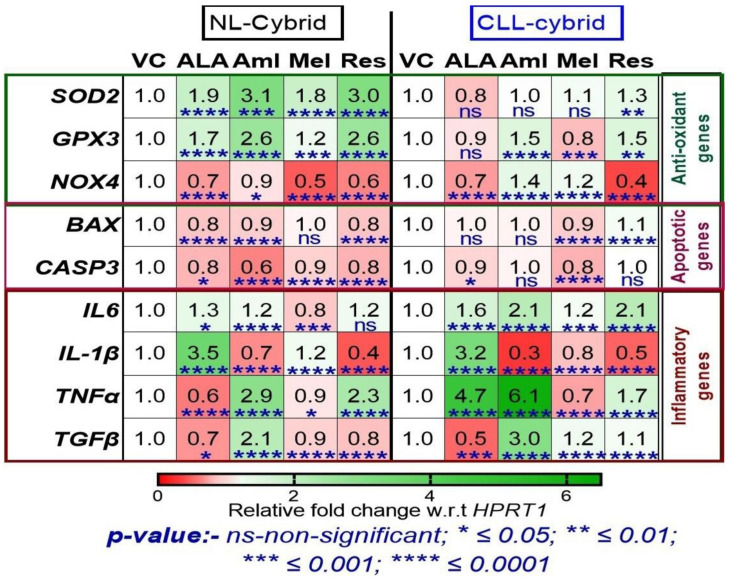
Heat map representation of treatment with alpha lipoic acid (ALA), amla (Aml), melatonin (Mel), and resveratrol (Res) on the mRNA expression of genes related to antioxidant, apoptotic, and inflammation using quantitative real-time polymerase chain reaction (qPCR) in the cybrid derived from age-matched control (NL) and chronic lymphoblastic leukemia (CLL) cybrids. Data are shown in the form of relative fold change with respect to housekeeping gene HPRT1 with respect to vehicle control and were analyzed by one-way ANOVA test. ns represents non-significant * indicates *p*-value *≤* 0.05; ** *<* 0.01; *** *<* 0.001; **** *<* 0.0001.

Antioxidant gene expression: The expression of *SOD2* and *GPX3* increased with ALA (1.9-fold change, *p* < 0.0001; 1.7-fold change, *p* < 0.0001), amla (3.1-fold change, *p* < 0.001; 2.6-fold change, *p* < 0.0001), melatonin (1.8-fold change, *p* < 0.0001; 1.2-fold change, *p* < 0.001), and resveratrol (3-fold change, *p* < 0.0001; 2.6-fold change, *p* < 0.0001) treatment, while decreasing the expression of *NOX4* by less than a one-fold change in all treatments in the NL cybrids compared with the vehicle-treated sample (Figure 5). Interestingly, the expression levels of *SOD2*, *GPX3*, and *NOX4* were either unaffected or slightly reduced in the CLL cybrid in response to the treatment with ALA (0.8-fold change, ns; 0.9-fold change, ns; 0.7-fold change, *p* < 0.0001), amla (1-fold change, ns; 1.5-fold change, *p* < 0.0001; 1.4-fold change, *p* < 0.0001), melatonin (1.1-fold change, ns; 0.8-fold change, *p* < 0.001; 1.2-fold change, *p* < 0.0001), and resveratrol (1.3-fold change, *p* < 0.01; 1.5-fold change, *p* < 0.01; 0.4-fold change, *p* < 0.0001), while there was a slight increase or decrease in the expression of *NOX4* in the CLL cybrid in response to the treatment with ALA (0.7-fold change, *p* < 0.0001), amla (1.4-fold change, *p* < 0.0001), melatonin (1.2-fold change, *p* < 0.0001), and resveratrol (0.4-fold change, *p* < 0.0001).

Apoptotic gene expression: We observed in NL cybrids that supplement-alone treatment with ALA (0.8-fold change, *p* < 0.0001), amla (0.9-fold change, *p* < 0.0001), and resveratrol (0.9-fold change, *p* < 0.0001) significantly reduced *BAX* expression (*p* < 0.0001) but there is no change in the expression of *BAX* in response to melatonin treatment (Figure 5). All of the supplements reduced *CASP3* expression levels; ALA (0.8-fold change, *p* < 0.05), amla (0.6-fold change, *p* < 0.0001), melatonin (0.6-fold change, *p* < 0.0001), and resveratrol (0.8-fold change, *p* < 0.0001) compared with the vehicle-treated samples (Figure 5). Interestingly, in the CLL cybrid, the expression of *BAX* was unaffected by treatment with ALA and amla (Figure 5). The BAX levels decreased in response to melatonin (0.9-fold change, *p* < 0.0001) but increased with resveratrol (1.1-fold change, *p* < 0.0001) in CLL cybrids compared with the vehicle-treated cells. The *CASP3* expression was reduced after treatment with ALA (0.9-fold change, *p* < 0.05) and melatonin (0.8-fold change, *p* < 0.0001).

Inflammatory gene expression: Next, we observed that supplement-alone treatment increased the expression levels of *IL6* with ALA (1.3-fold change, *p* < 0.05) and amla (1.2-fold change, *p* < 0.0001) in the NL cybrids, while there were decreased expression levels of *IL6* with melatonin (0.8-fold change, *p* < 0.0001) but no significant change with resveratrol treatment in the NL cybrid (Figure 5). Similarly, in the CLL cybrids, there was a significant increase in the expression of *IL6* in response to the treatment with ALA (1.6-fold change, *p* < 0.0001), amla (2.1-fold change, *p* < 0.0001), melatonin (1.2-fold change, *p* < 0.001), and resveratrol (2.1-fold change, *p* < 0.0001) (Figure 5).

Interestingly, there were increases and decreases in the expression levels of *IL1β* in both the NL and CLL cybrids. The significant expression levels of *IL1β* increased in response to ALA (3.5-fold change, *p* < 0.0001) and melatonin (1.2-fold change, *p* < 0.0001) in the NL cybrid, while treatment with amla (0.7-fold change, *p* < 0.0001) and resveratrol (0.4-fold change, *p* < 0.0001) significantly decreased the expression levels of *IL1β* in the NL cybrid (Figure 5). Similarly, in the CLL cybrid the ALA (3.2-fold change, *p* < 0.0001) significantly increased the expression levels of *IL1β*, whereas the other treatments significantly reduced the levels: amla (0.3-fold change, *p* < 0.0001), melatonin (0.8-fold change, *p* < 0.0001), and resveratrol (0.5-fold change, *p* < 0.0001) (Figure 5).

In the NL cybrids, we observed that supplement-alone treatment with ALA (0.6-fold change, *p* < 0.0001) and melatonin (0.9-fold change, *p* < 0.05) had significant decreases in the expression levels of *TNFα*, whereas there were significant increases in the expression levels of *TNFα* levels in response to treatment with amla (2.9-fold change, *p* < 0.0001) and resveratrol (2.3-fold change, *p* < 0.0001). In the CLL cybrids in response to melatonin (0.7-fold change, *p* < 0.0001), there were significant decreases in the expression levels of the *TNFα* levels. In contrast, in response to ALA (4.7-fold change, *p* < 0.0001), amla (6.1-fold change, *p* < 0.0001), and resveratrol (1.7-fold change, *p* < 0.0001), there were increased expression levels of *TNFα* (Figure 5). 

Next, we found significant increases of *TGFβ* levels in response to amla (2.1-fold change, *p* < 0.0001) in the NL cybrid and (three-fold change, *p* < 0.0001) and in the CLL cybrid (Figure 5). In contrast, in response to ALA, the NL cybrids showed decreased levels by 0.7-fold change (*p* < 0.0001), and the CLL cybrid had a 0.5-fold change (*p* < 0.0001) in the expression levels of *TGFβ*. NL cybrids showed decreased levels of *TGFβ* in response to melatonin (0.9-fold change, *p* < 0.0001) and resveratrol (0.8-fold change, *p* < 0.0001). In contrast, the CLL cybrids showed a significant increase in *TGFβ* in response to melatonin (1.2-fold change, *p* < 0.0001) and resveratrol (1.1-fold change, *p* < 0.0001) (Figure 5).

### 2.7. Effect of in-Combination Treatment of Ibrutinib with Mitochondrial Targeted Nutraceuticals on the Expression of Antioxidant, Apoptotic, and Inflammatory Genes in the Patient-Derived CLL and NL Cybrids

**Figure 6:**
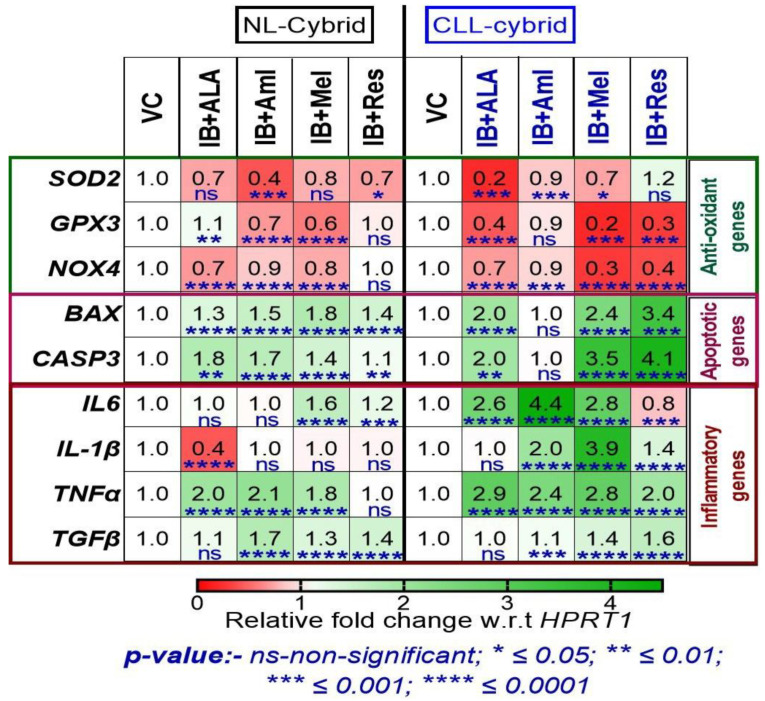
Heat map representation of treatment with alpha lipoic acid (ALA), amla (Aml), melatonin (Mel), and resveratrol (Res) on the mRNA expression of genes related to antioxidant, apoptotic, and inflammation using quantitative real-time polymerase chain reaction (qPCR) in the cybrid derived from age-matched control (NL) and chronic lymphoblastic leukemia (CLL) cybrids. Data are shown in the form of relative fold change with respect to housekeeping gene HPRT1 with respect to vehicle control and were analyzed by one-way ANOVA test. ns represents not significant * indicates *p*-value *≤* 0.05; ** *<* 0.01; *** *<* 0.001; **** *<* 0.0001.

Antioxidant gene expression: Next, we wanted to investigate the effect of combining mitochondria-targeting nutraceuticals (ALA, amla, melatonin, and resveratrol) with ibrutinib on the expression of genes related to antioxidants (Figure 6). In the NL cybrid, Ibr + Amla (Ibr + Amla, 0.4-fold change, *p* < 0.001) and Ibr + Resv (Ibr + Resv, 0.7-fold change, *p <* 0.05) significantly decreased the expression of *SOD2*, while there was no significant change in the expression levels of SOD2 in response to Ibr + ALA (Ibr + ALA) and Ibr + Mel (Ibr + Mel). The CLL cybrid also had lower expression of *SOD2* after exposure to the combinations Ibr + ALA (0.2-fold change, *p* < 0.001), Ibr + Amla (0.9-fold change, *p* < 0.001), and Ibr + Mel (0.7-fold change, *p <* 0.05). There was no significant change in the expression of *SOD2* in response to treatment to Ibr + Resv (1.2-fold change, ns).

In the NL cybrid cells, Ibr + Amla (0.7-fold change, *p* < 0.0001) and Ibr + Mel (0.6-fold change, *p* < 0.0001) significantly decreased the expression of *GPX3*, while the Ibr + ALA (1.1-fold change, *p <* 0.01) treatment significantly increased the expression of *GPX3* levels. There was no change in the *GPX3* levels after the Ibr + Resv treatment (Figure 6). In the CLL cybrid, there were reduced *GPX3* levels in response to Ibr + ALA (0.4-fold change, *p* < 0.0001), Ibr + Mel (0.2-fold change, *p* < 0.001), and Ibr + Resv (0.3-fold change, *p* < 0.001). There was no significant *GPX3* change in response to Ibr + Amla treatment (Figure 6).

In NL cybrid cells, treatment with Ibr + ALA (0.7-fold change, *p* < 0.0001), Ibr + Amla (0.9-fold change, *p* < 0.0001), and Ibr + Mel (0.8-fold change, *p* < 0.0001) significantly decreased the expression of *NOX4*, while in response to Ibr + Resv treatment, there was no significant change in the expression of *NOX4* (Figure 6). Interestingly, in the CLL cybrid in response to Ibr + ALA (0.7-fold change, *p* < 0.0001), Ibr + Amla (0.9-fold change, *p* < 0.001), Ibr + Mel (0.3-fold change, *p* < 0.0001), and Ibr + Resv (0.4-fold change, *p* < 0.0001), there were decreased *NOX4* expression levels (Figure 6).

Apoptotic gene expression: As shown in Figure 6, in the NL cybrids, the expression of *BAX* and *CASP3* increased in response to ibrutinib plus each of the supplements. *BAX* levels were as follows: Ibr + ALA (1.3-fold change, *p* < 0.0001), Ibr + Amla (1.5-fold change, *p* < 0.0001), Ibr + Mel (1.8-fold change, *p* < 0.0001), and Ibr + Resv (1.4-fold change, *p* < 0.0001). *CASP3* levels were as follows: Ibr + ALA (1.8-fold change, *p <* 0.01), Ibr + Amla (1.7-fold change, *p* < 0.0001), Ibr + Mel (1.4-fold change, *p* < 0.0001), and Ibr + Resv (1.1-fold change, *p* < 0.01) in the NL cybrid (Figure 6).

Interestingly, in the CLL cybrid, treatment with Ibr + ALA (two-fold change, *p* < 0.0001), Ibr + Mel (2.4-fold change, *p* < 0.0001), and Ibr + Resv (3.4-fold change, *p* < 0.001) significantly increased *BAX* expression, whereas there was no change in response to Ibr + Amla treatment (Figure 6) Similarly, Ibr + ALA (two-fold change, *p* < 0.01), Ibr + Mel (3.5-fold change, *p* < 0.0001), and Ibr + Resv (4.1-fold change, *p* < 0.01) significantly increased the *CASP3* expression, but there was no significant change in the expression of *CASP3* in response to Ibr + Amla.

Inflammatory gene expression: Next, we observed in NL cybrids, in response to Ibr + Mel (1.6-fold change, *p* < 0.0001), and Ibr + Resv (1.2-fold change, *p* < 0.0001), there were significantly increased expression levels of *IL6*. There was no change in the expression levels of *IL6* with resveratrol treatment (Figure 6). In the CLL cybrids, there was significantly increased expression of *IL6* in response to the Ibr + ALA (2.6-fold change, *p* < 0.0001), Ibr + Amla (4.4-fold change, *p* < 0.0001), and Ibr + Mel (2.8-fold change, *p* < 0.0001). In contrast, Ibr + Resv (0.8-fold change, *p* < 0.001) significantly decreased the expression of the CLL cybrid (Figure 6).

Interestingly, the expression levels of *IL1β* were significantly decreased in response to Ibr + ALA (0.4-fold change, *p* < 0.0001) in the NL cybrid, while in response to Ibr + Amla, Ibr + Mel, and Ibr + Resv, there were no changes in the expression levels of *IL1β* in the NL cybrid (Figure 6). In contrast, the CLL cybrid showed significantly increased expression levels of *IL1β* in response to Ibr + Amla (two-fold change, *p* < 0.0001), Ibr + Mel (3.9-fold change, *p* < 0.0001), and Ibr + Resv (1.4-fold change, *p* < 0.0001). There was no significant change in the expression of *IL1β* in response to Ibr + ALA in the CLL cybrid (Figure 6).

We observed that in-combination treatment of Ibr + ALA (two-fold change, *p* < 0.0001), Ibr + Amla (2.1-fold change, *p* < 0.0001), and Ibr + Mel (1.8-fold change, *p* < 0.0001) showed significant increases in the expression levels of *TNFα*, whereas no significant change was seen in response to Ibr + Resv in the NL cybrids (Figure 6). In the CLL cybrid cells, there were significant increases of *TNFα* expression levels in response to Ibr + ALA (2.9-fold change, *p* < 0.0001), Ibr + Amla (2.4-fold change, *p* < 0.0001), Ibr + Mel (2.8-fold change, *p* < 0.0001), and resveratrol (two-fold change, *p* < 0.0001) (Figure 6). 

In the NL cybrids, we found significant increases in the expression of *TGFβ* in response to Ibr + Amla (1.7-fold change, *p* < 0.0001), Ibr + Mel (1.3-fold change, *p* < 0.0001), and Ibr + Resv (1.4-fold change, *p* < 0.0001), whereas no significant change was seen in response to Ibr + ALA treatment in the NL cybrid (Figure 6). Similar significant increases in *TGFβ* expression were observed in the CLL cybrid (Figure 6) in response to Ibr + Amla (1.1-fold change, *p* < 0.0001), Ibr + Mel (1.4-fold change, *p* < 0.0001), and Ibr + Resv (1.6-fold change, *p* < 0.0001.

## 3. Discussion

**Figure 7:**
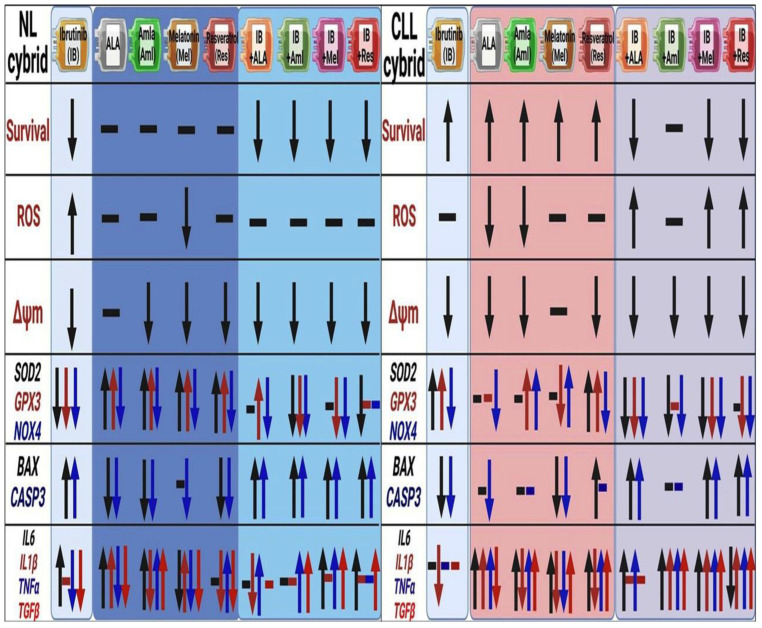
Schematic illustration of change in the level of survival, reactive oxygen species (ROS), mitochondrial membrane potential, gene expression related to antioxidant, apoptotic, and inflammation in the NL and CLL cybrids. All the comparisons are measured with respect to the vehicle control of the respective cybrid.

In this study, we generated cybrid cell lines from a CLL patient and an age-matched control subject. Using these transmitochondrial cybrids, we demonstrated that cells containing the CLL mitochondria were more resistant to ibrutinib treatment than cells with age-matched control mitochondria. However, as shown in Figure 3, ibrutinib in combination with ALA, melatonin, and resveratrol (mitochondria-targeted nutraceuticals) significantly sensitized the cells to the ibrutinib, making the percent survival lower than the ibrutinib-alone-treated cells. Moreover, the combination of ALA, melatonin, and resveratrol with ibrutinib increased cellular ROS production, mitochondrial membrane potential, and expression of apoptotic genes and decreased the expression of antioxidant genes in the CLL cybrids but not in the NL cybrids (Figure 7).

Our study showed that the mtDNA from the CLL patient’s cybrid was defined as the N haplogroup, which is an ancestral line that descended directly from haplogroup L3. Early members of this group lived in the eastern Mediterranean and western Asia [34]. Mutations in the D-loop region have been reported in human neurodegenerative and ophthalmologic disorders, cardiovascular diseases, and cancers including leukemic. Some researchers identified associations between SNPs m.15784T>C, m.16185C>T, and m.16399A>G SNPs located in the mtDNA D-loop region that have been reported in breast cancer [35,36]. The m.73A>G, m.152T>C, m.189A>G, m.16223C>T, and m.16390G>A SNPs located in the mtDNA D-loop region have been reported in breast cancer [35,36]. The present study identified two private SNPS in the CLL cybrid’s mtDNA regions: m.2866A>T (no rs#, RNR2) and m.13635T>C (no rs#, MT-ND5), which were not linked to any known pathogenesis. 

A review of the literature shows that pathways involved with the mitochondria are inextricably linked to chemoresistance, which is a survival strategy employed by cancer cells in response to apoptotic stimulation [37]. Cancer cells rely heavily on mitochondria for a variety of functions, including metabolism, calcium signaling, mitochondrial dynamics regulation, and resistance to oxidative stress [37]. Mitochondrial plasticity is involved in many stages of tumor development, including chemoresistance. In fact, recent research suggests that chemoresistant ovarian cancer cells gain an advantage over their non-resistant counterparts when their mitochondria undergo a bioenergetic switch to oxidative metabolism at the same time [38]. Our findings that CLL mitochondria are resistant to ibrutinib treatment while the NL cybrids are sensitive are in line with these studies.

While it is not always clear what causes oxidative stress in cancer patients, it has been recognized that excessive levels of ROS can be produced by tumor cells and cells that interact with tumors. Compared with normal B cells, CLL cells have higher mitochondrial respiration rates, consistent with elevated levels of mitochondria-derived ROS. This, in turn, can cause increased oxidative stress, which can make CLL cells resistant to chemotherapy [39]. Interestingly, in NL cybrid cells, the ibrutinib treatment in combination with ALA, amla, melatonin, and resveratrol did not significantly change the mean difference levels of ROS production compared with the vehicle-treated cells (Figure 7) and led to lower levels of ROS when compared with ibrutinib treatment alone. In contrast the same treatment in the CLL cybrid significantly increased the mean difference levels of ROS by 21% (Ibr + ALA), 35% (Ibr + Mel), and 21% (Ibr + Res), compared with the ibrutinib-alone-treated CLL cells. One can speculate that the CLL cybrid combination treatment produces more ROS than the ibrutinib treatment alone, which may sensitize the CLL cybrid to accelerated cell death.

Diminished mitochondrial membrane potential (ΔΨm) is a hallmark of mitochondrial dysfunction [40]. It has been observed that cancer cells have a more negative net transmembrane potential (ΔΨm) compared with normal cells, which may be caused by alterations in mitochondrial bioenergetics [41]. Our findings show that combining Ibr + ALA, melatonin, and resveratrol improves the mitochondrial health, as evidenced by higher ΔΨm levels compared with the CLL cells treated with ibrutinib alone. This was surprising because lower ΔΨm levels are often associated with elevated apoptosis, but our gene expression studies showed that the CLL cybrid treated with the combination of ibrutinib plus ALA, melatonin, and resveratrol had two-fold or greater levels of *BAX* and *CASP3*, which are pro-apoptosis genes. As a result of higher mitochondria membrane potential, there may be improved oxidative phosphorylation, which would lead to significantly increased levels of ROS production. This may result in CLL cybrid sensitization to ibrutinib in combination with ALA, melatonin, and resveratrol rather than individual ibrutinib treatment as seen in Figure 3.

The hypersensitivity of normal cells to ROS makes them vulnerable to carcinogenic effects if they are not properly protected by antioxidant mechanisms. In contrast, chemoresistant cancer cells have antioxidant mechanisms (glutathione, SOD, catalase, and others) that are up-regulated, protecting them from ROS [42]. Our data imply that elevated expression of *SOD2* and *GPX3*, as seen in the CLL cybrids (Figure 6), may be reducing ROS production and contributing to the CLL cybrid resistance to ibrutinib treatment.

Targeting BAX proteins and permeabilizing the mitochondrial outer membrane induces apoptosis in chemotherapy. Pro-apoptogenic substances from the mitochondrial intermembrane space are released into the cytosol by mitochondrial outer membrane permeabilization, activating a caspase cascade that kills the cancer cell [43,44]. Our results raise the possibility that the decreased expression of apoptotic genes, *BAX* (0.4-fold) and *CASP3* (0.5-fold), contributes to CLL cybrids’ resistance to ibrutinib treatment by increasing cell survival. Conversely, the expression of apoptotic genes was not as greatly affected by treatment with mitochondria-specific nutraceuticals alone (range 0.8-fold to 1.1-fold change). In contrast, ibrutinib treatment with ALA, melatonin, and resveratrol significantly up-regulates the expression of *BAX* (range 2-fold to 3.4-fold) and *CASP3* (2-fold to 3.9-fold) in CLL cybrid cells.

Some of the negative effects of chemotherapy include an increase in pro-inflammatory cytokines [45,46], which can contribute to cancer-related fatigue and pro-inflammatory cytokines that play a pivotal role in chemotherapy-related side effects [47]. Many chemotherapeutics, such as cisplatin, etoposide, ibrutinib, paclitaxel, topotecan, and vinblastine, cause cell death by inducing DNA damage, which causes nuclear and mitochondrial DNA leakage into the cytosol and activates cGAS-STING signaling, which is part of innate immunity [48,49]. Our findings show that in-combination treatment up-regulates the pro-inflammatory genes in CL cybrids to higher levels than seen in the NL age-matched control cybrid cells. This would result in more inflammation and untoward side effects in the CLL patients compared with what might be found in cells with normal mitochondria. 

Our results show that using mitochondrial-targeted nutraceuticals with ibrutinib increased cellular ROS production, mitochondrial membrane potential, and expression of apoptotic genes and decreased the expression of antioxidant genes in CLL cybrids. These results suggest that mitochondria are critical players in cancer cell survival because they serve as the bioenergetic and biosynthetic hub that coordinates cellular respiration, ETC, apoptosis, antioxidant signaling, and redox homeostasis. Cancer drug resistance, as an adaptive strategy used by cancer cells to survive stress conditions, is inextricably linked to mitochondrial-related pathways. Indeed, emerging evidence strongly suggests that resistant tumor cells have high mitochondrial respiration and OXPHOS status. As a result, targeting mitochondria represents a promising cancer treatment avenue and chemoresistance-overcoming strategy.

## 4. Materials and Methods

### 4.1. Generation of Patient-Derived Cybrid

**Figure 8:**
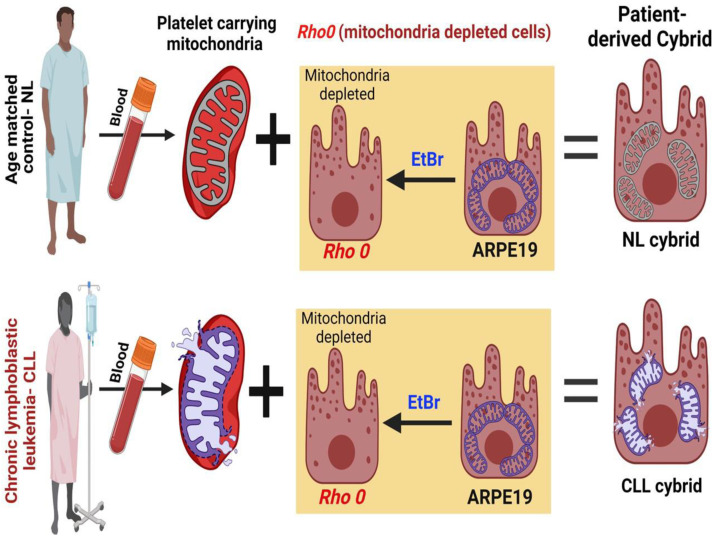
Schematic illustration of the creation of cybrids from the age-matched control (NL) subject and chronic lymphoblastic leukemia (CLL) patient.

Transmitochondrial cybrids were created by fusing a mitochondrial DNA-deficient ARPE-19 (*Rho0*) cell line with platelets isolated from either chronic lymphoblastic leukemia (CLL) patients or age-matched control, normal (NL) subjects (Figure 8). Ten milliliters of peripheral blood were collected in tubes containing 3.2% sodium citrate from age-matched NL and CLL patients. To isolate platelets, blood was centrifuged, and final platelet pellets were resuspended in Tris-buffered saline. ARPE-19 cells *Rho0* cells (mitochondria DNA-deficient) were created by passaging ARPE-19 cells in low doses (50 ng/mL) of ethidium bromide several times and (0.025 g) uridine supplement in the media. The *Rho0* cells were fused with platelets via polyethylene glycol fusion, and the newly formed cybrids were then cultured to passage 5 in media containing DMEM-F12, 10% dialyzed fetal bovine serum, 100 unit/mL penicillin, 100 µg/mL streptomycin, 2.5 µg/mL fungizone, 50 µg/mL gentamicin, and 17.5 mM glucose. 

### 4.2. Whole mtDNA Genome Sequencing of the CLL Cybrid

The sequencing method was a modified version of one used by Zaragoza et al. [50]. We were able to quantify the SNPs that are homoplasmic, heteroplasmic, haplogroup defining, private (non-haplogroup defining), and unique (those not listed in www.MitoMap.org) across the entire mitochondrial genome of the CLL subject using this sequencing method. The PCR for whole mtDNA genome sequencing was performed in two parts using a high-fidelity PCR system (FailSafe TM PCR System, Lucigen, Madison, WI) and two sets of primers. Part A used primers hmtL569 [AACCAAACCCCAAAGACAC] and hmtH12111 [AAACCCGGTAATGAT-GTCGG], while Part B used primers hmtL11727 [GCCCACGGGCTTACATC] and hmtH1405 [ATCCACCTTCGACCCTTAAG] (Integrated DNA Technologies, Inc., Coralville, IA). The PCR products were run on a 1% agarose gel, and the unincorporated primers and dNTPs were eliminated enzymatically in a single step (ExoSAP-IT, Thermo Fisher Scientific, Pittsburgh, PA, USA). Samples were sent to ELIM BioPharm for sequencing using internal primers (ELIM Biopharm, Hayward, CA, USA). The sequencing results were analyzed using DNA variant analysis software (Mutation Surveyor, SoftGenetics, State College, PA, USA). The mtDNA sequences were compared with the classification from www.hmtvar.uniba.it (accessed on 12 October 2022).

### 4.3. Identification of mtDNA Haplogroup in the CLL Cybrid

The DNA was extracted from cell pellets from the CLL patient-derived cybrids using the Purelink^TM^ Genomic DNA Mini Kit (Invitrogen, Thermo Fisher Scientific, Carlsbad, CA, USA), according to the manufacturer’s instructions. Mitochondrial DNA profiles were determined through sequencing, as has been previously described [51]. In general, the mtDNA haplogroup profile for the cybrids was compared with the sequence of the blood samples to verify the successful introduction of the mitochondria with the personalized mtDNA into the cybrid cell line.

### 4.4. Cell Survival 

NL and CLL cybrids were plated in a 96-well plate at a density of 10,000 cells per well in 100 μL of culture media. After 24 h plating, the cells were treated for 48 h with ibrutinib, ALA, amla, melatonin, and resveratrol at concentrations of 10 μM, 1 mM, 300 μg, 1 mM, and 100 μM, respectively. After the completion of the treatment, the cell metabolism was measured using the colorimetric MTT assay kit according to the manufacturer’s protocol (MTT Cell Viability Assay Kit, Biotium, Fremont, CA, USA). A minimal of three replicates were tested per sample. Each experiment was repeated three times.

### 4.5. Reactive Oxygen Species (ROS) Assay

The NL and CLL cybrids were plated in a 96-well plate at a density of 10,000 cells per well in 100 μL of culture media. Twenty-four hours after plating, the cells were treated for 48 h with ibrutinib, ALA, amla, melatonin, resveratrol, and in combination at concentrations of 10 μM, 1 mM, 300 μg, 1 mM, and 100 μM, respectively. Following the treatment process, the cells were exposed to the fluorescent H_2_DCFDA (2,7-dichlorodihydrofluorescin diacetate) (Invitrogen-Molecular Probes, Carlsbad, CA, USA) dye. The ROS levels were measured using a fluorescence plate reader (Gemini XPS Microplate Reader, Molecular Devices, Sunnyvale, CA, USA) at an excitation wavelength of 492 nm and emission wavelength of 520 nm. A minimum of three replicates were tested per sample. Each experiment was repeated three times.

### 4.6. JC-1 Mitochondrial Membrane Potential (ΔΨm) Assay

The NL and CLL cybrids were plated in a 96-well plate at a density of 10,000 cells per well in 100 μL of culture media. After 24 h plating, the cells were treated for 48 h with ibrutinib, ALA, amla, melatonin, resveratrol, and in combination at concentrations of 10 μM, 1 mM, 300 μg, 1 mM, and 100 μM, respectively. Following the treatment process, the cells were exposed to 5,5′,6,6′-tetrachloro-1,1′,3,3′-tetraethylbenzimidazolylcarbocyanineiodide (Biotium, Hayward, CA, USA) for 15 min. The mitochondrial membrane potential was then measured with a fluorescence plate reader (Gemini XPS Microplate Reader, Molecular Devices, Sunnyvale, CA, USA) by measuring the fluorescence for red (excitation 550 nm and emission 600 nm) and green (excitation 485 nm and emission 545 nm). Intact mitochondria with normal ΔΨm appeared red, whereas impaired cells with diminished ΔΨm were green. The ratio of red/green was used for analysis. A lower ratio corresponded to higher apoptotic/dead cell number. A minimum of three replicates were tested per sample. Each experiment was repeated three times.

### 4.7. RNA Extraction, cDNA Synthesis, and qRT-PCR 

Patient-derived NL and CLL cybrids were cultured and incubated for 24 h in six-well plates. Twenty-four hours after plating, the cells were treated 48 h with ibrutinib, ALA, amla, melatonin, resveratrol, and in combination at concentrations of 10 μM, 1 mM, 300 μg, 1 mM, and 100 μM, respectively. After the completion of 48 h of treatment, RNA extraction was performed on both the NL and CLL cybrid cells using an RNA isolation kit (Purelink TM RNA Kit, Ambion, Thermo Fisher Scientific, Waltham, MA, USA) as per the manufacturer’s protocol. The RNA quantity and purity were measured on a Nanodrop 1000 spectrophotometer (Thermo Fisher Scientific). An OD 260/280 of 1.8–2.0 indicated the purity of RNA devoid of protein contamination. Concentrations of 500 ng/μL RNA were reverse transcribed into cDNA using a SuperScript^TM^ VILO^TM^ IV cDNA Synthesis Kit (Thermo Fisher Scientific). The mRNA levels of mitochondrial biogenesis genes were determined by quantitative reverse transcription-PCR (qRT-PCR) using PowerUp SYBR Green Master Mix (Thermo Fisher Scientific). Reactions were carried out in a final volume of 10 μL. KiCqStart^®^ SYBR^®^ green primers (Sigma, St. Louis, MO, USA) were used to examine the expression of *SOD2* (Ref ID# NM_000636), *GPX3* (Ref ID# NM_002084), *NOX4* (Ref ID# NM_001143836), *BAX* (Ref ID# NM_004324), *CASP3* (Ref ID# NM_004346), *IL6* (Ref ID# NM_000600), *IL-1β* (Ref ID# NM_000576), *TNF-α* (Ref ID# NM_000594), and *TGF-β* (Ref ID# NM_003238). The housekeeper gene was Hypoxanthine Phosphoribosyl Transferase 1 (*HPRT1*, Ref ID# NM_ NM_000194). Each PCR reaction was followed by continuous melt curve analysis. No template controls (NTCs) were also included in each PCR run to assess contamination. All samples were run in triplicate. Data analyses were performed after normalization to the reference gene to calculate a fold change value using the ∆∆Ct method, which was calculated by subtracting the difference between the threshold cycles (Cts) of the target gene and the housekeeper gene (*HPRT1* gene). The fold change was calculated using the following formula: fold change = 2^−∆∆Ct^. A fold change value ≥ 1 was considered as ‘up-regulation’, whereas a fold change value < 1 was considered as ‘down-regulation’ of the gene.

### 4.8. Statistical Analysis

GraphPad Prism 9 software was used to perform statistical analyses on all quantitative data (GraphPad Software, Inc., San Diego, CA, USA). All of the analyses were compared with the vehicle control group. Unless otherwise specified, comparisons between experimental groups were made using one-way ANOVA followed by Sidak’s multiple comparisons method.

## 5. Conclusions

To conclude, mitochondria from CLL patients are more likely to become resistant to ibrutinib treatment than those from age-matched control patients while ibrutinib combined with mitochondria-targeted nutraceuticals, especially ALA, melatonin, and resveratrol, significantly sensitizes CLL cybrids by increasing the cellular reactive oxygen species (ROS) production, mitochondrial membrane potential, and expression of apoptotic genes and decreasing the expression of antioxidant genes.

## Data Availability

All data relevant to the study are included in the article.

## Supplementary Materials

The supporting information can be downloaded at https://www.mdpi.com/article/10.3390/ijms241311025/s1.
