# Supplementary material for: Using Human ‘Personalized’ Cybrids to Identify Drugs/Agents That Can Regulate Chronic Lymphoblastic Leukemia Mitochondrial Dysfunction
Source: Int J Mol Sci. 2023 Jul 3;24(13):11025. doi: 10.3390/ijms241311025 (PMC10341973; doi:10.3390/ijms241311025)
Supplement: Supplementary file 1 [file ijms-24-11025-s001.zip › Supplemetary Figure S1.pdf]

Supplementary file

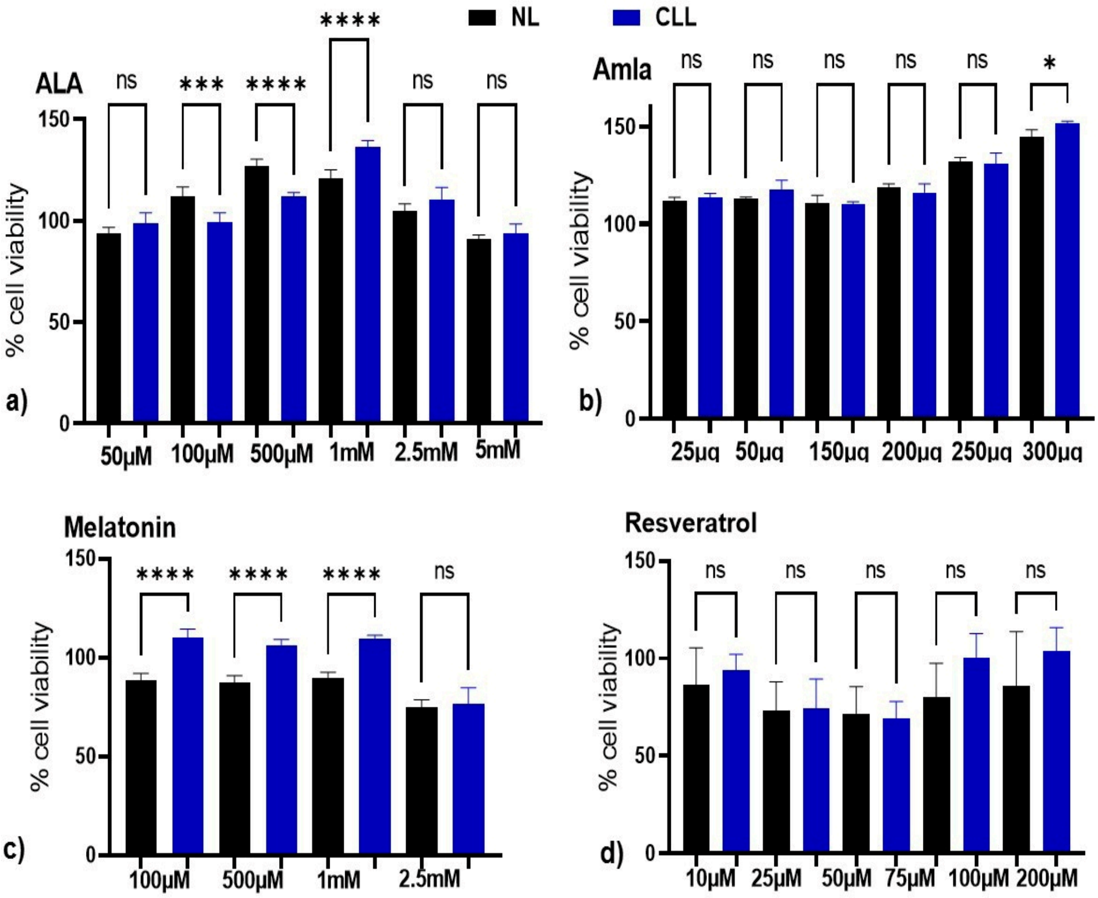

Supplementary Figure S1. Effect of mitochondria-targeted nutraceuticals, i.e., alpha lipoic acid (ALA), amla (Aml), melatonin (Mel), and resveratrol (Res), on the survival of the cybrid derived from age-matched control (NL) and chronic lymphoblastic leukemia (CLL) cybrids with different concentrations to determine the optimum concentration. (a) Impact of alpha lipoic acid (ALA) treatment on the survival of NL cybrid and CLL cybrid using MTT assay. (b) Impact of amla (Aml)treatments on the survival of CLL cybrids using MTT assay. (c) Impact of melatonin (Mel) treatments on the viability of CLL cybrids using MTT assay. (d) Impact of resveratrol (Res) treatments on the viability of CLL cybrids using MTT assay. Percent cell viabilities of respective ALA, Aml, Mel, and Res were measured with respect to the vehicle control of NL and CLL cybrids. Data shown as mean ± SD were analyzed by one-way ANOVA test. * indicates *p*-value ≤ 0.05; ***< 0.001; **** < 0.0001.
