# Supplementary material for: Using Human ‘Personalized’ Cybrids to Identify Drugs/Agents That Can Regulate Chronic Lymphoblastic Leukemia Mitochondrial Dysfunction
Source: Int J Mol Sci. 2023 Jul 3;24(13):11025. doi: 10.3390/ijms241311025 (PMC10341973; doi:10.3390/ijms241311025)
Supplement: Supplementary file 1 [file ijms-24-11025-s001.zip › Supplemetary Table S1.pdf]

Supplementary table

Supplementary table S1: SNPs found by Sanger sequencing in CLL cybrid

Loci: MT-	SNP	AA change	Functional Loci: MT	rs#	CLL-cybrid	Related pathogenesis	Literature (PMID#)	Variants
DLOOP	73 A>G	-	Regulatory	rs869183622	ND	Oncolytic adenoma, Ewing sarcoma, Breast cancer, Lung cancer	28104394, 28245399, 24603941, 20055758	ND
DLOOP	152 T>C	-	Regulatory	rs117135796	L2	Bladder cancer, Lung cancer, Ovarian carcinoma, Liver cancer, Nasopharyngeal carcinoma, Aging	26639157	ND
RNR1	1438 A>G	-	rRNA	rs2001030	Non-H	Hearing loss, Polymorphic, Cardiomyopathy, Benign	28104394, 18325329, 11938495, 20055758, 23826813, 19535463	ND
RNR1	1598 G>A	-	rRNA	rs3135027	N1b	Hearing loss, benign	20064630, 23826813	Homoplasmic, Heteroplasmic
RNR2	1703 C>T	-	rRNA	rs28527344	N1b1	Mito encephalopathy	2102678	Heteroplasmic
RNR2	1719 G>A	-	rRNA	rs3928305	N1'5	Ischemic stroke	18593462, 26883569, 11938495, 20055758, 25650108	Homoplasmic, Heteroplasmic
RNR2	2639 C>T	-	rRNA	ND	N1b	ND	Unavailable	ND
RNR2	2706 A>G	-	rRNA	rs2854128	Non-H	Cardiomyopathy, Hyperlactemia, Anaemia, Asthenia, Hearing loss	28104394, 11024191, 11938495, 27217714, 20055758, 25650108, 28674062, 27323166	ND
**RNR2**	**2866 A>T**	**-**	**rRNA**	**ND**	**PVT-Unique**	ND	Unavailable	**ND**
ND1	3921 C>A	Synonymous (Ser, aa position 205)	Coding	ND	N1b1	ND	Unavailable	ND
ND2	4735 C>A	Thr→Asn, aa position 89	Coding	ND	N1b1b	ND	Unavailable	ND
ND2	4769 A>G	Synonymous (Met, aa position 100)	Coding	rs3021086	Non-H	Protective in Alzheimer disease (AD), Dementia, Mitochondrial myopathy	28104394, 18325329	ND

ND2	4917 A>G	Asn→Asp, aa position 150	Coding	rs28357980	N1b1b	Leber Hereditary Optic Neuropathy (LHON), Alzheimer disease (AD)	17152068, 1370613	Homoplasmic
ND2	4960 C>T	Ala→Val, aa position 164	Coding	ND	N1b1	ND	Unavailable	Homoplasmic
ND2	5471 G>A	Synonymous (Thr, aa position 334)	Coding	ND	N1b	ND	Unavailable	ND
CO1	7028 C>T	Synonymous (Ala, aa position 375)	Coding	rs2015062	Non-H	Mental retardation, Strabismus, Ischemic stroke	27033601, 26883569, 11938495, 20055758, 18593462	ND
CO2	8251 G>A	Synonymous (Gly, aa position 222)	Coding	rs3021089	N1b	ND	Unavailable	Homoplasmic
ATP8	8472 C>T	Pro → Leu, aa position 36	Coding	ND	N1b1	Autism	Unavailable	Homoplasmic
ATP6	8836 A>G	Met → Val, aa position 104	Coding	ND	N1b	Leber Hereditary Optic Neuropathy (LHON)	29670542, 23508290, 30273650	Homoplasmic, Heteroplasmic
ND3	10238 T>C	Synonymous (Ile, aa position 60)	Coding	rs28358275	N1	ND	Unavailable	Homoplasmic
ND4	11719 G>A	Synonymous (Gly, aa position 320)	Coding	rs2853495	Non-R	Leber Hereditary Optic Neuropathy (LHON), Neuropathy	28104394, 24603941, 11938495, 27323166	ND
ND4	11928 A>G	Asn → Ser, aa position 390	Coding	ND	N1b1b	ND	Unavailable	Heteroplasmic
ND5	12501 G>A	Synonymous (Met, aa position 55)	Coding	rs28397767	N1	ND	Unavailable	Homoplasmic
ND5	12705 C>A	Ile → Met, aa position 123	Coding	ND	Non-R	Mobility impairment	30335417	ND
ND5	12822 A>G	Synonymous (Ala, aa position 162)	Coding	ND	N1b1	ND	Unavailable	ND
ND5	13635 T>C	Synonymous (Gly, aa position 433)	Coding	ND	PVT-ND5 (Syn)	Likely benign	**Unavailable**	**ND**

ND5	13710 A>G	Synonymous (Ala, aa position 458)	Coding	rs200295632	N1b1b	ND	Unavailable	Homoplasmic
ND6	14581 T>C	Synonymous (Val, aa position 31)	Coding	ND	N1b1b1	ND	Unavailable	ND
CYB	14766 C>T	Thr → Ile, aa position 7	Coding	rs193302980	Non-H	Breast cancer, Normal tension glaucoma (NTG)	28104394, 22475488, 11938495, 24448266, 22223843, 19682603	ND
CYB	15326 A>G	Thr → Ala, aa position 194	Coding	rs2853508	ND	Breast cancer, Ataxia, Septo optical dyslasia	28104394, 25132006, 24102627, 11811990, 11938495, 22659402, 24448266, 19682603	ND
DLOOP	16145 G>A	-	Regulatory	rs41419246	N1b1	ND	Unavailable	Homoplasmic
DLOOP	16223 C>T	-	Regulatory	rs2853513	ND	Mitochondrial disease, Breast cancer, Bladder cancer, Lung cancer, Nasopharyngeal carcinoma, Stomach cancer, Liver cancer	28104394, 24603941, 15240908, 20055758, 22930374	ND
DLOOP	16390 G>A	-	Regulatory	rs41378955	N1b	Parkinson’s disease, Bladder cancer, Lung cancer, Breast cancer	27217714, 26883569, 22930374	Homoplasmic, Heteroplasmic
